# Neddylation and Its Target Cullin 3 Are Essential for Adipocyte Differentiation

**DOI:** 10.3390/cells13191654

**Published:** 2024-10-05

**Authors:** Hongyi Zhou, Vijay Patel, Robert Rice, Richard Lee, Ha Won Kim, Neal L. Weintraub, Huabo Su, Weiqin Chen

**Affiliations:** 1 Departments of Physiology, Medical College of Georgia at Augusta University, Augusta, GA 30912, USA; 2 Department of Surgery, Medical College of Georgia at Augusta University, Augusta, GA 30912, USA; vijpatel@augusta.edu (V.P.); rrice@augusta.edu (R.R.); ricklee@augusta.edu (R.L.); 3 Department of Medicine, Medical College of Georgia at Augusta University, Augusta, GA 30912, USA; hkim3@augusta.edu (H.W.K.); nweintraub@augusta.edu (N.L.W.); 4 Vascular Biology Center, Medical College of Georgia at Augusta University, Augusta, GA 30912, USA; hsu@augusta.edu

**Keywords:** adipogenesis, neddylation, post-translational modification, metabolism, obesity

## Abstract

The ongoing obesity epidemic has raised awareness of the complex physiology of adipose tissue. Abnormal adipocyte differentiation results in the development of systemic metabolic disorders such as insulin resistance and diabetes. The conjugation of NEDD8 (neural precursor cell expressed, developmentally downregulated 8) to target protein, termed neddylation, has been shown to mediate adipogenesis. However, much remains unknown about its role in adipogenesis. Here, we demonstrated that neddylation and its targets, the cullin (CUL) family members, are differentially regulated during mouse and human adipogenesis. Inhibition of neddylation by MLN4924 significantly reduced adipogenesis of 3T3-L1 and human stromal vascular cells. Deletion of NAE1, a subunit of the only NEDD8 E1 enzyme, suppressed neddylation and impaired adipogenesis. Neddylation deficiency did not affect mitotic cell expansion. Instead, it disrupted CREB/CEBPβ/PPARγ signaling, essential for adipogenesis. Interestingly, among the neddylation-targeted CUL family members, deletion of CUL3, but not CUL1, CUL2, or CUL4A, largely replicated the adipogenic defects observed with neddylation deficiency. A PPARγ agonist minimally rescued the adipogenic defects caused by the deletion of NAE1 and CUL3. In conclusion, our study demonstrates that neddylation and its targeted CUL3 are crucial for adipogenesis. These findings provide potential targets for therapeutic intervention in obesity and metabolic disorders.

## 1. Introduction

Adipose tissue is the most flexible tissue in the body due to constant remodeling in adipocyte size and number [1]. Abnormal adipocyte differentiation or turnover will result in obesity, predisposing to various systemic metabolic disorders such as insulin resistance and diabetes. Adipogenesis is orchestrated by a general two-step transcriptional program [2] with CCAAT/enhancer-binding protein β (CEBPβ) and CEBPδ induced transiently to initiate adipocyte differentiation [3,4]. Activation of CEBPβ/δ then leads to the second-wave induction of two master transcription factors, peroxisome proliferator-activated receptor-γ (PPARγ) and CEBPα, which in turn promote terminal adipocyte differentiation [2,5,6] and maintain mature adipocyte function [7]. cAMP-response element-binding protein (CREB) is a central upstream transcription factor that controls the activation of the CEBPβ/PPARγ cascade [8,9,10]. Despite this well-established paradigm, upstream signaling governing this axis is poorly understood. 

NEDD8 (neural precursor cell expressed, developmentally downregulated 8) is an 81 amino acid ubiquitin-like protein involved in the post-translational modification process known as neddylation [11]. This process attaches one or multiple NEDD8 moieties to the lysine residues of target proteins, mediated by a cascade of specific enzymes: the E1 activating enzyme (a heterodimer consisting of NAE1 and UBA3), the E2 conjugating enzymes (UBE2M/UBE2F), and E3 ligases [12]. Neddylation regulates a variety of cellular processes, such as the ubiquitin-proteasome system (UPS)-mediated protein degradation, cell cycle control, differentiation, and autophagy [13,14,15]. Dysregulation of neddylation has been linked to several pathological conditions, including cancer [16,17] and heart failure [18]. In 3T3-L1 cells, NEDD8 deficiency significantly impairs adipocyte differentiation by affecting PPARγ neddylation and protein stability [19]. MLN4924 (MLN), a specific inhibitor of NAE1-mediated neddylation [20], has been demonstrated to suppress adipogenesis and prevent high-fat diet (HFD)-induced obesity [19]. These findings suggest that neddylation plays a critical role in adipose tissue development. However, the specific targets of neddylation involved in adipogenesis need to be further investigated to fully understand the underlying mechanisms. 

Cullin (CUL) family members, including CUL1, CUL2, CUL3, CUL4A, CUL4B, CUL5, CUL7, and CUL9, are among the best-characterized targets of neddylation. Neddylation of CULs promotes the assembly and activation of approximately 400 CUL–RING ubiquitin (Ub) ligases (CRLs), which ubiquitylate key cellular regulators and target them for degradation via the UPS [15]. Interestingly, only scattered evidence has shown the involvement of different CULs in adipogenesis and obesity [21], highlighting the need for more significant efforts to determine the role of CULs in adipose biology. CUL3, in particular, contains a conserved neddylation site at its C-terminal lysine 762 (K762) [22]. By serving as a molecular scaffold, neddylated CUL3 facilitates the interaction between catalytic modules (a Ub E2 enzyme and the RING finger domain protein, RBX1) and target recognition modules (adaptors with a BTB domain) [23,24]. CUL3-based CRL3 complexes are vital for various cellular processes, including differentiation, stress responses, and embryonic and organ development [25]. Loss-of-function mutations in CUL3 are linked to familial hyperkalemic hypertension [26,27], and CUL3 knockdown in human Lisa2 preadipocytes has been shown to impair adipocyte differentiation [28]. Despite these insights, the detailed role of CUL3 and other CULs in adipogenesis remains to be fully explored. 

In this study, we first examined the expression profiles of neddylation and its targeted CULs during the differentiation of mouse and human adipocytes. Employing the CRISPR/Cas9 technique, we next specifically deleted the regulatory subunit of the only neddylation E1 enzyme, NAE1, and various CULs in 3T3-L1 cells and assessed the impact of these deletions on adipocyte differentiation. Our findings indicate that deficiencies in neddylation and its target CUL3, not CUL1, CUL2, and CUL4A, significantly impair CREB/CEBPβ/PPARγ signaling, thereby hindering adipogenesis. Moreover, although a PPARγ agonist can minimally rescue this adipogenic deficiency, it is insufficient to fully compensate for the lack of neddylation and CUL3.

## 2. Materials and Methods

### 2.1. Cell Culture, CRISPR/Cas9 Gene Targeting, Lentivirus Production and Infection, and 3T3-L1 Adipocyte Differentiation

If not specified, all cell culture media and antibiotics were obtained from Fisher Scientific (Pittsburg, PA, USA), and all drugs were obtained from Sigma-Aldrich ( Sr. Louis, MO, USA). All cells were cultured in a 37 °C incubator with 5% CO_2_. All-in-one pLenti-CRISPR V2 empty vector or gRNAs against murine Nae1, Cul1, Cul2, Cul3, and Cul4a were obtained from GenScript Biotech (Piscataway, NJ, USA). Specific gRNA sequences against each gene were presented in Table 1. Lentiviral particles were produced in HEK293T cells (ATCC, Manassas, VA, USA, CRL-3216™) as previously described [29]. The 3T3-L1 cells (ATCC, CL-173) were maintained in Dulbecco’s modified Eagle’s medium (DMEM) containing 10% calf bovine serum (BCS, ATCC^®^ 30-2030™) and penicillin–streptomycin (Pen/Strep). Proliferating 3T3-L1 preadipocytes were infected with lentivirus supernatants combined with fresh DMEM complete medium at a 1:1 ratio in the presence of 8 μg/mL polybrene (Santa Cruz Biotechnology, Santa Cruz, CA, USA, NC9840454). Cells were then selected with 2 µg/mL puromycin (MilliporeSigma, Burlington, MA, USA, 540411) for at least four days before being cultured to confluence for adipocyte differentiation. Given the proliferative differences caused by NAE1 deletion in 3T3-L1 cells, we plated different numbers of cells to ensure all cells reach confluence the next day for adipocyte differentiation to avoid the confounding effects of proliferation. Two days after confluence, 3T3-L1 differentiation was induced by culturing in DMEM media with 10% fetal bovine serum (FBS, Thermo Scientific Cat# SH3091003HI) and Pen/Strep in the presence of standard hormones (DMI, dexamethasone; IBMX, 3-isobutyl-1-methylxanthine, and insulin) as we previously described [30].

### 2.2. Isolation of Mouse Embryonic Fibroblasts (MEF) for Adipocyte Differentiation

C57BL/6J mice were housed at a 12 h light/12 h dark cycle with ad libitum access to food and water. MEFs were isolated from 12.5 to 14.5-day-old embryos from C57BL/6J mice as described [31]. Isolated cells were then subjected to differentiation using a standard hormone protocol in the presence of 1 µM pioglitazone as described [32].

### 2.3. Isolation of Human Stromal Vascular Cells from Adipose Tissue and Adipocyte Differentiation

We obtained de-identified human mediastinal adipose tissues from patients undergoing cardiothoracic surgeries as surgical waste. Tissues were minced and digested in HBSS buffer with 2% BSA and 1 mg/mL collagenase type IV (Worthington Biochemical Corporation, Lakewood, NJ, USA, LS004188) at 37 °C for 40 to 60 min. The digest was filtered through a 250 μm pore-size mesh followed by 500× *g* for 10 min to separate the stromal vascular cells (SVCs) from the floating adipocytes. SVC pellets were resuspended in RBC lysis buffer (SCBT, Santa Cruz, CA, USA, NC0536296) at 37 °C for 10 min. The pellets were then resuspended in human preadipocyte growth media (Cell Applications Inc., San Diego, CA, USA, 811-500). Cells were cultured to confluence for two days and subjected to differentiation as described [33]. Briefly, cells were cultured in cell differentiation media consisting of DMEM/F12, 15 mM HEPES, 25 mM NaHCO3, 100 units/mL Pen/Strep, 33 µM d-biotin, 17 µM pantothenate, 100 nM dexamethasone, 100 nM insulin, 1 µM pioglitazone, 0.5 mM IBMX, 2 nM T3, and 10 µg/mL transferrin). After 6–7 days of induction, cells were changed to maintenance media consisting of DMEM/F12 with Pen/Strep, d-biotin, pantothenate, 10 nM insulin, and 10 nM dexamethasone until fully differentiated. The medium was changed every 2–3 days. 

### 2.4. FACS Analysis 

About 2 × 10^6^ cells were washed and fixed using 70% ethanol overnight. Pelleted cells were then washed twice with cold PBS. Cells were then resuspended in 500 µL propidium iodide (PI)/Triton X-100 staining solution: 10 mL of 0.1 % (*v*/*v*) Triton X-100 in PBS with 0.2 mg/mL DNAse-free RNAse A and 20 µg/mL PI and incubated at 37 °C for 15 min. Data were acquired using FACSCalibur (Becton Dickinson, Franklin Lakes, NJ, USA) under 605 nm lasers and analyzed according to the Abcam protocol.

### 2.5. Triglyceride (TG) Contents and Oil Red O (ORO) Staining

Cells were harvested directly in PBS buffer containing 1% triton X-100. An aliquot of homogenates was used to measure the intracellular TG content using an Infinity Triglyceride assay kit (Thermofisher Scientific, West Columbia, SC, USA, TR-22421). Data were normalized to the amount of cellular protein as determined by a PierceTM Bradford Protein Assay Kit (Thermofisher Scientific, West Columbia, SC, USA, 23200). Oil-red O staining was performed as described previously (47). Images were photographed with a camera or under a microscope. 

### 2.6. Real-Time Quantitative PCR

Total RNA was isolated from cells with TRIzolTM (Thermofisher Scientific, 15-596-018) and reverse-transcribed using MLV-V reverse transcriptase (ThermoFisher Scientific, 28025013) with random primers (ThermoFisher Scientific, 48190011). Real-time quantitative RT-PCR was performed using iTaq Univer SYBR Green Supermix (Bio-Rad, Hercules, CA, USA, 1725125) on the AriaMX system (Agilent Technologies, Santa Clara, CA, USA). Data were normalized to two housekeeping genes (Ppia and Rplp0) based on the Genorm algorithm (RRID: SCR_006763) and expressed as fold changes. Table 2 lists the RT-PCR primer sequences for genes that were analyzed. 

### 2.7. Immunoblot Analysis

Cells were homogenized and lysed in lysis buffer containing 25 mM Tris-HCl (pH 7.4), 150 mM NaCl, 2 mM EDTA, 1% Triton X-100, and 10% glycerol with freshly added protease inhibitor cocktail (Sigma, P8340) and phosphatase inhibitor (Fisher Scientific, Pittsburg, PA, USA, AAJ61022AA). The protein concentration was determined by the PierceTM Bradford Protein Assay Kit (Thermofisher, 23200). Western blots were carried out according to the standard protocol. The blots were visualized using the ECL chemiluminescence system by Amersham Imager 600, followed by densitometry qualification using ImageQuantTL v8.2.0 (Cytivia). Antibodies are listed in Table 3. 

### 2.8. Statistical Analysis

Quantitative data were presented as means ± SEM. Experiments were performed from at least two independent cohorts. Differences between groups were examined for statistical significance with unpaired *t*-tests or by one-way or two-way ANOVA as dictated by the experiments using GraphPad Prism Software 10.2.2 (RRID: SCR_002798). A *p* value of less than 0.05 was considered statistically significant.

## 3. Results

### 3.1. Dysregulated Neddylation and Its Targets CULs during Mouse and Human Adipogenesis

**Figure 1:**
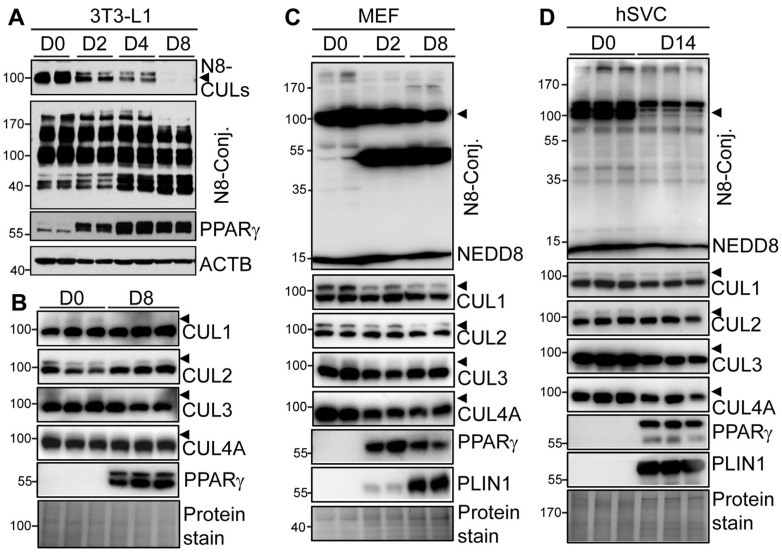
Neddylation and its Cullin family targets are differentially regulated during adipogenesis. (**A**,**B**) Protein expression in day (D) 0 preadipocytes, Day 2 differentiating, and Day 8 mature adipocytes during DMI-induced 3T3-L1 adipocyte differentiation. Three independent experiments. (**C**) Protein expression during different days of DMI+pioglitazone-induced MEF adipocyte differentiation. Two independent experiments. (**D**) Protein expression in day 0 human SVCs isolated from mediastinal adipose biopsy and 14 days (D14) after adipocyte differentiation. Representative data from four independent human donors were shown. Arrowheads indicated neddylated CULs detected by the NEDD8 antibody. Due to the complexity of the data, quantification of Western blots was not performed.

We first examined how neddylation is altered during adipogenesis of the widely used preadipocyte cell line, 3T3-L1. We found that the total level of NEDD8-conjugated CULs was reduced along the course of adipocyte differentiation. In contrast, the levels of NEDD8-conjugated non-cullin proteins were either increased or decreased in mature adipocytes (Figure 1A). Consistent with the reduced neddylated CULs, the neddylation of CUL1 and CUL2 was significantly reduced in day 8 mature adipocytes as compared to day 0 preadipocytes (Figure 1B). Screening of the currently commercial antibodies against CUL3 and CUL4 did not consistently detect the neddylated forms of CUL3 and CUL4 in preadipocytes and adipocytes. There were no significant changes in the expression of native CULs before and after 3T3-L1 differentiation (Figure 1B). Similar findings were also identified in MEF undergoing adipocyte differentiation, as evidenced by decreased neddylated CULs (i.e., CUL1 and CUL2) and altered non-CUL protein neddylation (Figure 1C). However, we also detected the reduced level of native CUL4A and free NEDD8 during MEF differentiation (Figure 1C). We further isolated human stromal vascular cells (hSVCs) from discarded mediastinal adipose tissue and subjected them to adipocyte differentiation. Again, compared to day 0 hSVCs, neddylated CULs and free NEDD8 in differentiated day 14 adipocytes were largely reduced when probed against NEDD8 (Figure 1D). The neddylated non-CUL proteins were mostly downregulated with some upregulated in human differentiated adipocytes (Figure 1D). The levels of neddylated CUL1 and CUL2 were consistently reduced, whereas the expression of native CUL3 and CUL4A was even decreased in human adipocytes (Figure 1D). In all experiments, the robust upregulation of PPARγ and/or PLIN1 proteins was used as mature adipocyte markers (Figure 1A–D). Therefore, our data suggest altered neddylation levels in CULs and non-CUL proteins during human and mouse adipocyte differentiation.

### 3.2. Pharmacologic Inhibition of Neddylation Blocks Mouse and Human Adipogenesis

**Figure 2:**
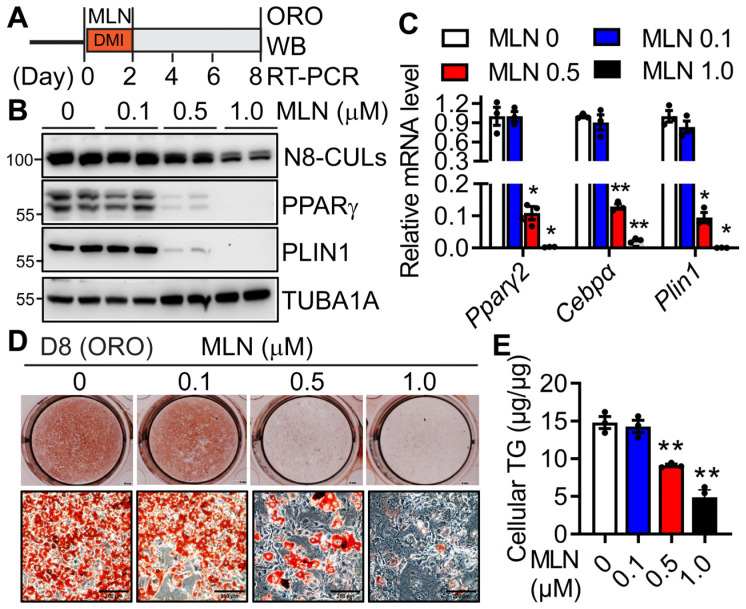
Inhibition of neddylation by MLN4924 blocks 3T3-L1 adipogenesis. (**A**) 3T3-L1 preadipocytes were treated with vehicle or indicated doses of MLN4924 (MLN) from Day 0-2, together with standard DMI hormone cocktails. On Day 8 (D8) of adipocyte differentiation, cells were harvested for oil-red O (ORO) staining, Western blot (WB), and RT-PCR. (**B**) Western blot and (**C**) RT-PCR of adipocyte marker proteins (*n* = 3). *, *p* < 0.05; **, *p* < 0.005 vs vehicle-treated MLN 0 group. Two-way ANOVA with Tukey’s multiple comparisons test. (**D**) Day 8 3T3-L1 adipocytes were stained with ORO (scale bar: 200 µm), and (**E**) cellular TG contents were quantified by enzymatic assays (*n* = 3). Data were normalized to cellular proteins. **, *p* < 0.005 vs. vehicle-treated MLN 0 group. One-way ANOVA with Dunnett’s multiple comparison test. Three independent experiments.

To understand the role of neddylation in adipogenesis, we treated 3T3-L1 cells with MLN4924 (MLN), a specific neddylation inhibitor [20], during the first two days of adipocyte differentiation concurrently with the standard DMI (dexamethasone, IBMX and insulin) hormone cocktail (Figure 2A). At day 8, we found that MLN could still reduce neddylated CULs (Figure 2B) dose-dependently. MLN treatment repressed the mRNA (Figure 2C) and protein (Figure 2B) expressions of the adipocyte marker genes (PPARγ, CEBPα, and PLIN1) in a concentration-dependent manner. Its treatment also dose-dependently blunted the accumulation of intracellular neutral lipids, as evidenced by oil red O staining (Figure 2D) and quantitative measurement of intracellular TGs (Figure 2E). Notably, MLN’s impairment on adipogenesis was not due to its widely reported role in proliferation or apoptosis [34], since 0.5 µM MLN resulted in no differences in cell numbers during the first 48 h of treatment compared to vehicle-treated cells (Supplementary Figure S1). These data indicate the essential role of the neddylation pathway in adipogenesis during the first two days of adipocyte differentiation.

**Figure 3:**
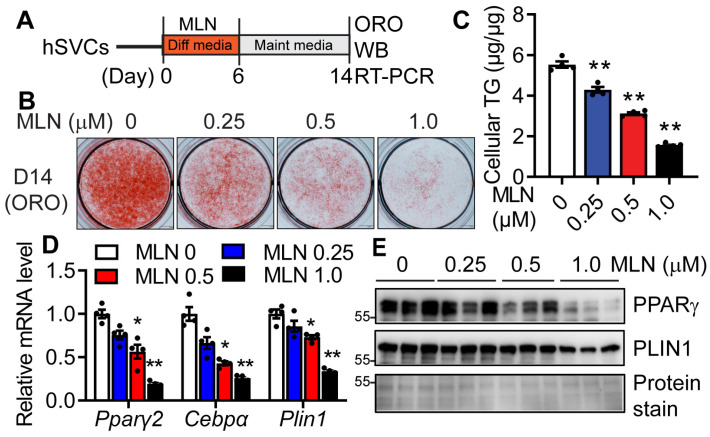
Inhibition of neddylation by MLN4924 blocks adipocyte differentiation of human stromal vascular cells. (**A**) hSVCs were treated with vehicle (0 µM) or indicated doses (0.25, 0.5, and 1.0 µM) of MLN4924 (MLN) from Day 0-6, together with human adipocyte differentiation media. Cells were then changed to human adipocyte maintenance media until Day 14 for oil red O (ORO) staining, Western blot (WB), and RT-PCR. (**B**) ORO staining and (**C**) cellular TG contents after normalization to cellular proteins (*n* = 4). **, *p* < 0.005 vs. vehicle-treated MLN 0 group. One-way ANOVA with Dunnett’s multiple comparison test. (**D**) RT-PCR analysis of adipocyte differentiation markers (Pparγ2, Cebpα, and Plin1) (*n* = 4). *, *p* < 0.05; **, *p* < 0.005 vs. vehicle- treated MLN 0 group. Two-way ANOVA with Tukey’s multiple comparisons test. (**E**) Western blot of adipocyte markers (PPARγ and PLIN1) at D14 mature adipocytes. Three independent experiments.

We further subjected hSVCs to adipocyte differentiation in the presence or absence of MLN with the differentiation media during the first 6 days of differentiation (Figure 3A). Again, by day 14 when vehicle-treated hSVCs fully differentiated into adipocytes, MLN treatment dose-dependently inhibited adipocyte differentiation as evidenced by reduced intracellular TG accumulation (Figure 3B,C) and suppressed mRNA (Figure 3D) and protein expressions (Figure 3E) of adipocyte marker genes (*PPARγ*, *CEBPα*, and *PLIN1*). Together, these data suggest that pharmacological inhibition of neddylation by MLN during the early stage of adipocyte differentiation leads to a detrimental effect on the formation of mature adipocytes.

### 3.3. Genetic Deletion of NAE1 Blocks Adipogenesis

**Figure 4:**
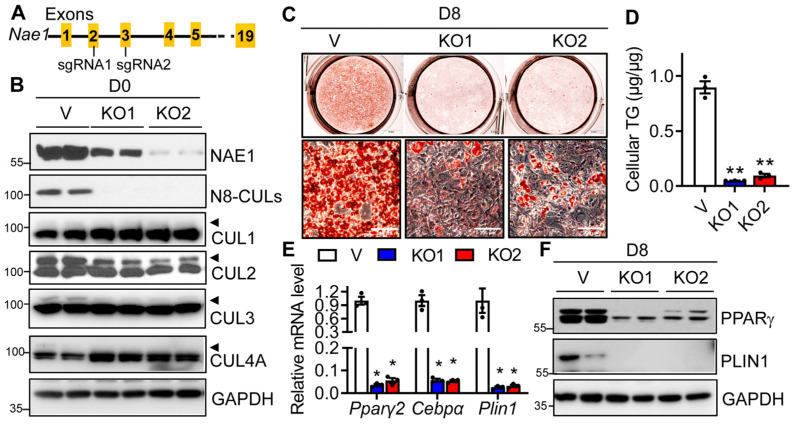
CRISPR/Cas9-mediated NAE1 deletion in 3T3-L1 cells blocks adipogenesis. (**A**) Targeting strategy of specific gRNA1 and gRNA2 against mouse Nae1 exons. (**B**) Western blot in Day 0 (D0) 3T3-L1 cells stably infected with lentiviruses generated from pLentiCRISPR v2 vector-targeted control cells (V) and pLentiCRISPR v2 expressing gRNA1 (NAE1^KO1^, KO1) and gRNA2 (NAE1^KO2^, KO2) against Nae1. (**C**) Representative pictures of ORO staining (scale bar = 200 µm), (**D**) cellular TG content (normalized to protein level) (*n* = 3). **, *p* < 0.005 vs. vector-targeted cells. One-way ANOVA with Dunnett’s multiple comparison test. (**E**) RT-PCR (*n* = 3) and (**F**) Western blot analyses of adipocyte marker proteins. *, *p* < 0.05 vs. vector-targeted cells. Two-way ANOVA with Tukey’s multiple comparisons test. (**C**–**F**) Performed in Day 8 (D8) V and NAE1^KO1^ and NAE1^KO2^ 3T3-L1 cells. Three independent experiments.

We further used the CRISPR/Cas9 approach to delete the *Nae1* gene, a subunit of the only NEDD8 E1 enzyme (Figure 4A), and we examined its impact on neddylation and adipocyte differentiation in 3T3-L1 cells. We confirmed the deletion of NAE1 at the protein level by Western blot, which led to significantly reduced total neddylated CULs, including neddylated CUL1, 2, 3, and 4A at day 0 before adipocyte differentiation (Figure 4B). While vector-targeted control cells can fully differentiate into adipocytes, both lines of NAE1-knockout (NAE1^KO1^ and NAE1^KO2^) cells demonstrated almost completely abolished ORO staining of neutral lipids (Figure 4C) and reduced cellular TG content (Figure 4D). mRNA (Figure 4E) and protein expressions (Figure 4F) of adipocyte markers were also drastically repressed in NAE1^KO^ cells. These data suggest that NAE1 deletion causes neddylation deficiency, which inhibits adipocyte differentiation in vitro. Together, our data demonstrate the crucial role of neddylation in adipogenesis.

### 3.4. Neddylation Deficiency Does Not Affect Mitotic Clone Expansion but Impairs CREB/CEBPβ/PPARγ Signaling

**Figure 5:**
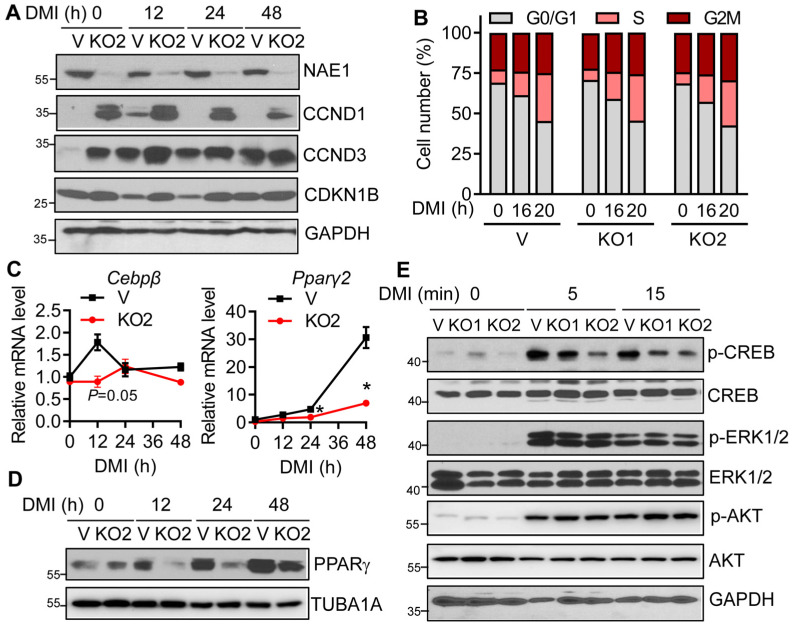
Neddylation deficiency impairs CREB/CEBPβ/PPARγ signaling during early adipogenesis. (**A**) Cell cycle protein expression at indicated hours (h) after DMI induction in vector-targeted control (V) and NAE1 gRNA2-knockout (NAE1^KO2^, KO2) cells. (**B**) FACS analysis of cell population at different cell cycle stages after DMI treatment for indicated times (0, 16, and 20 h) in V, NAE1 gRNA1-knockout (NAE1^KO1^, KO1), NAE1^KO2^ 3T3-L1 cells. Two independent experiments. (**C**) RT-PCR (*n* = 3) and (**D**) Western blot in NAE1^KO2^ 3T3-L1 cells at 0, 12, and 24 h after DMI induction. *, *p* < 0.05. Multiple unpaired t-tests with the Holm–Sidak method. (**E**) Western blot to analyze signaling pathways in response to DMI induction at 0, 5, and 15 min (mins) in V, KO1, and KO2 3T3-L1 preadipocytes. Data are representative of three independent experiments.

Neddylation is essential for cell cycle progression by activating CUL-based CRLs, such as SKP1/CUL1/F-box protein (SCF)-like Ub ligase complex [15,35,36]. Mitotic clonal expansion (MCE) during the first two days of differentiation is a prerequisite for terminal adipocyte differentiation [37]. Indeed, we found SCF-like Ub ligases-targeted CCND1 (cyclin D1), CCND3 (cyclin D3), and CDKN1B (p27) were upregulated at day 0 before DMI treatment in NAE1^KO^ preadipocytes. In vector cells, DMI triggered transient upregulation of CCND1 and CCND3 at 12 h and downregulation of CDKN1B at 12 and 24 h, suggesting mitotic clonal expansion through cell cycle progression (Figure 5A). Interestingly, CCND1, CCND3, and CDKN1B were aberrantly upregulated at all times after DMI stimulation in NAE1^KO^ cells. Such regulation did not exert a significant effect on cell cycle progression from G0/G1 to S and further to G2M in two distinct NAE1^KO^ cells after DMI induction (Figure 5B and Supplemental Figure S2). Therefore, aberrant regulation of both cell cycle activators and inhibitors in NAE1^KO^ cells exerted no effect on MCE during the first day after adipogenic induction. 

Transient upregulation of CEBPβ at the early-phase differentiation is required for subsequent PPARγ activation and adipogenesis [3,4]. We found that basal mRNA expression of CEBPβ was comparable in Vector and NAE1^KO2^ cells at day 0. However, while there was a significant upregulation of CEBPβ mRNA at 12 h after adipogenic induction in vector cells, such regulation was blunted in NAE1^KO2^ cells (Figure 5C). Consequently, the increase in PPARγ in NAE1^KO2^ cells was attenuated at mRNA and protein levels starting from as early as 24 h post-DMI induction (Figure 5C and Figure 5D, respectively). 

DMI acutely activates CREB phosphorylation at Serine 133 and regulates its transcriptional activity to control CEBPβ expression [10]. cAMP-activated Protein Kinase A (PKA) directly mediates CREB phosphorylation upon DMI induction [38]. Interestingly, we found CREB phosphorylation was significantly dampened as early as five minutes after DMI induction in both NAE1^KO1^ and NAE1^KO2^ cells without altering total CREB expression, while insulin-mediated phosphorylation of ERK1/2 and AKT remained intact (Figure 5E). Collectively, our data suggest that neddylation acts on CREB/CEBPβ/PPARγ signaling to affect adipogenesis.

### 3.5. Deletion of CUL3, but Not CUL1, 2 and 4A Blocks Adipogenesis

**Figure 6:**
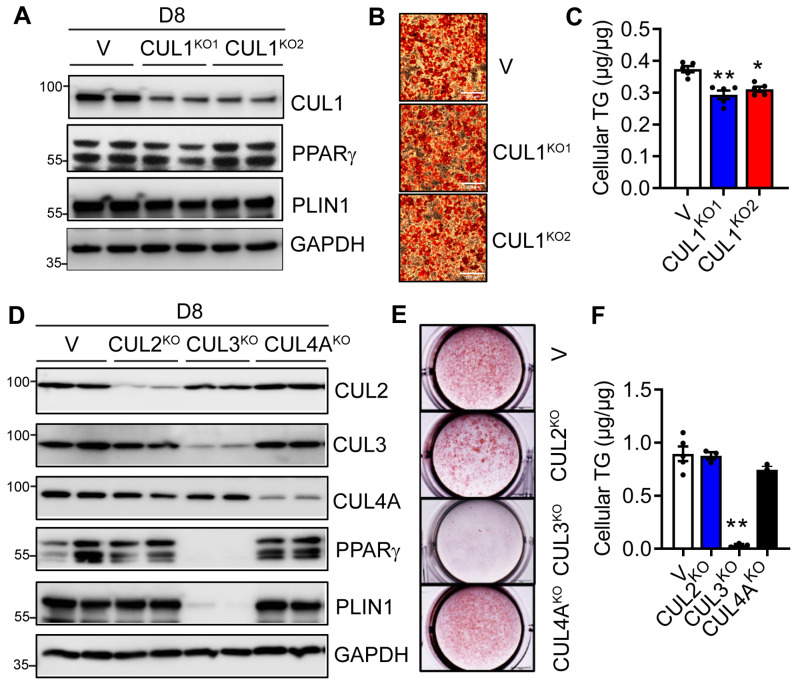
Knockout of CUL3, but not CUL1, 2, and 4A, in 3T3-L1 cells blocks adipogenesis. Vector-targeted control (V), CUL1 gRNA1–knockout (CUL1^KO1^), and gRNA2-knockout (CUL1^KO2^) 3T3-L1 preadipocytes were subjected to DMI-induced adipocyte differentiation. (**A**) Western blot, (**B**) representative pictures of oil red O (ORO) staining (scale bar = 200 µm), and (**C**) cellular TG contents quantified by enzymatic assays and normalized to protein levels (*n* = 5) at day 8 (D8) of adipocyte differentiation. *, *p* < 0.05; **, *p* < 0.005. One-way ANOVA with Dunnett’s multiple comparison test. (**D**–**F**) Control (V), CUL2 gRNA1–knockout (CUL2^KO^), CUL3 gRNA1-knockout (CUL3^KO^), and CUL4A gRNA1-knockout (CUL4A^KO^) 3T3-L1 preadipocytes were subjected to DMI-induced adipocyte differentiation. (**D**) Western blot analyses, (**E**) representative pictures of ORO staining (scale bar = 5 mm), and (**F**) cellular TG contents quantified by enzymatic assays and normalized to protein levels (*n* = 3–5) at Day 8. **, *p* < 0.005. One-way ANOVA with Dunnett’s multiple comparison test. Three independent experiments.

Next, we examined whether neddylation-targeted CULs are involved in adipocyte differentiation. We first generated two CUL1-knockout (CUL1^KO1^ and CUL1^KO2^) 3T3-L1 preadipocyte cell lines using two different gRNAs via the CRISPR/Cas9 approach. We confirmed the successful deletion of CUL1 in both cell lines at day 0. On day 8 of adipocyte differentiation, both cell lines still demonstrated an almost 100% deletion of CUL1 (Figure 6A). Interestingly, the deletion of CUL1 led to no differences in the expression of adipocyte marker proteins (Figure 6A). ORO staining revealed no apparent changes in the accumulation of neutral lipids (Figure 6B). However, quantitative cellular TG analyses showed very slight reductions in TG contents in both CUL1^KO1^ and CUL1^KO2^ adipocytes (Figure 6C). We further applied the same CRISPR/Cas9 approach to delete CUL2, CUL3, and CUL4A in 3T3-L1 preadipocytes individually and analyzed their adipogenic capacity. As shown in Figure 6D, the deletion of CUL3, but not CUL2 and CUL4A, affected the expression of adipocyte marker proteins on day 8 (Figure 6D). This was further confirmed by ORO staining and quantitative analysis of cellular TG contents, which demonstrated a completely blunted neutral lipid accumulation only in day 8 CUL3^KO^ 3T3-L1 cells (Figure 6E,F). Together, our studies demonstrate the crucial role of neddylation-targeted CUL3 in adipogenesis.

### 3.6. CUL3 Deletion Led to Impaired CREB/CEBPβ/PPARγ Signaling and Adipogenic Deficiency

**Figure 7:**
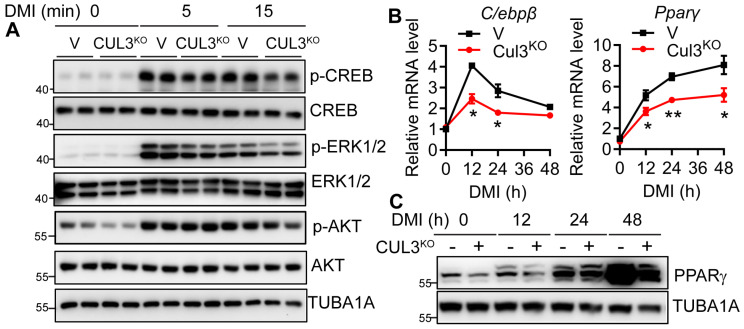
CUL3 deficiency also impairs CREB/CEBPβ/PPARγ signaling during adipocyte differentiation. (**A**) Western blot to analyze signaling pathways in response to DMI induction for the indicated 0, 5, and 15 min (mins) in vector-targeted control (V) and CUL3^KO^ 3T3-L1 preadipocytes. (**B**) RT-PCR (*n* = 3) and (**C**) Western blot in V and CUL3^KO^ 3T3-L1 cells at 0, 12, 24, and 48 h after DMI induction. *, *p* < 0.05; **, *p* < 0.005. Multiple unpaired t-tests with the Holm–Sidak method. Data are representative of three independent experiments.

To further examine whether CUL3 deletion led to a similar deficiency in the initial adipogenic signaling as NAE1 deletion, we analyzed the DMI-triggered CREB/CEBPβ/PPARγ signaling in CUL3^KO^ 3T3-L1 cells. Like NAE1^KO^ cells, CUL3^KO^ cells failed to initiate a normal CREB phosphorylation in response to DMI. Meanwhile, we observed a slight reduction in insulin-mediated ERK1/2 but not AKT phosphorylation in CUL3^KO^ cells (Figure 7A). Again, this was in line with the reduced mRNA expressions of *Cebpβ* and *Pparγ2* as early as 12 h after DMI induction in CUL3^KO^ cells (Figure 7B). Western blot further confirmed the reduced PPARγ protein starting from 12 h post-DMI induction in CUL3^KO^ cells (Figure 7C). These data emphasize that deficiency of neddylation and its target CUL3 causes similar deficiency in DMI triggered adipogenic signaling, leading to blunted adipogenesis.

### 3.7. RHOA/ROCK Inhibitor Fails to Restore, but Pioglitazone Minimally Rescues Adipogenesis in CUL3^KO^ and NAE1^KO^ Cells

**Figure 8:**
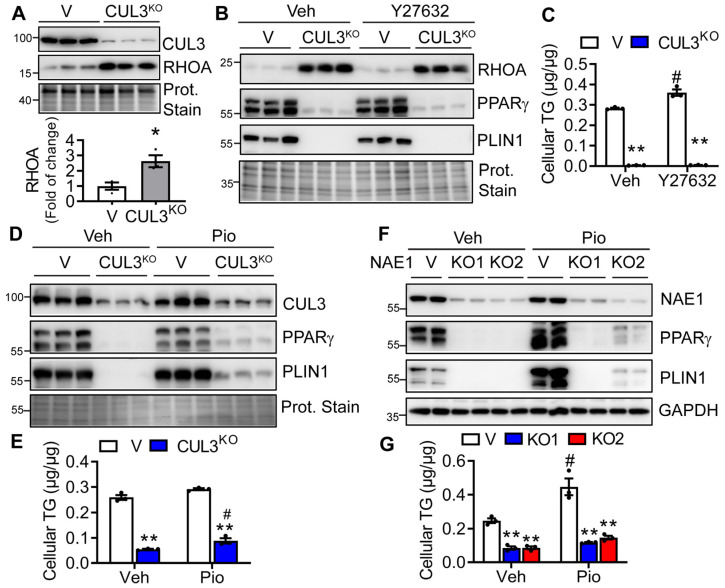
ROCK inhibitor fails, and pioglitazone minimally rescues the blunted adipogenesis caused by CUL3 and neddylation deficiency. (**A**) Western blot and quantification to analyze RHOA protein expression in Day 0 vector-targeted control (V) and CUL3^KO^ 3T3-L1 preadipocytes (*n* = 3). *, *p* < 0.05. Unpaired *t*-test. (**B**,**C**) V and CUL3^KO^ 3T3-L1 preadipocytes were subject to DMI-induced adipocyte differentiation in the presence of vehicle (Veh) or 10 µM Y27632 until day 8 for (**B**) Western blot analyses and (**C**) cellular TG contents (normalized to cellular proteins) (*n* = 3). (**D**,**E**) V and CUL3^KO^ 3T3-L1 cells and (**F**,**G**) V, NAE1^KO1^ (KO1), NAE1^KO2^ (KO2) 3T3-L1 cells were subject to DMI-induced adipocyte differentiation in the presence of vehicle (Veh) or 1 µM pioglitazone until Day 8 for Western blot analyses and cellular TG contents (normalized to cellular proteins) (*n* = 3). (**C**,**E**,**G**): **, *p* < 0.005 vs. V with the same treatment. #: *P* < 0.05 vs. the same cells treated with the vehicle. Two-way ANOVA with Tukey’s multiple comparisons test. Three independent experiments.

Previous studies have implicated the role of CRL3 in lipid droplet growth and the degradation of RHOA, regulating adipogenesis [28]. High RHOA/ROCK signaling suppresses PPARγ expression and actin polymerization, and, thus, adipogenesis [39]. CRL3^KEAP1^ was shown to regulate RHOA [28]. Indeed, we found elevated RHOA expression in CUL3^KO^ 3T3-L1 preadipocytes (Figure 8A). We next treated differentiating cells with RHOA/ROCK inhibitor Y27632 to test whether it can rescue the adipocyte differentiation in CUL3^KO^ cells. However, while Y27632 can slightly strengthen the adipocyte differentiation in vehicle-treated vector cells, it failed to rescue CUL3^KO^ adipogenesis to any extent as assessed by the lack of increase in the expression of adipocyte markers (PPARγ and PLIN1) (Figure 8B) as well as cellular TG content (Figure 8C). Since both CREB/CEBPβ as well as RHOA/ROCK signaling act on PPARγ to regulate adipogenesis, we further pursued whether treating differentiating cells with PPARγ agonist pioglitazone can rescue the adipocyte differentiation in CUL3^KO^ and NAE1^KO^ cells. To our surprise, treatment with pioglitazone had minimal effects on restoring adipocyte differentiation in both CUL3^KO^ (Figure 8D,E) and NAE1^KO^ (Figure 8E,F) cells, based on the very slight upregulation of PPARγ and PLIN1 as well as minimal restoration of lipid accumulation. Therefore, besides PPARγ, CUL3 and NAE1 deletion may also perturb other pathways to regulate adipocyte differentiation.

## 4. Discussion

In this study, we systematically characterized the crucial roles of neddylation and its well-known targets, the CUL family members, in regulating adipogenesis. We first confirmed that MLN4924, an inhibitor of neddylation, inhibits mouse and human adipogenesis in vitro, consistent with the previous report [19]. We generated NAE1^KO^ 3T3-L1 preadipocytes to further investigate neddylation deficiency on adipogenesis using the CRISPR/Cas9 technique. These NAE1^KO^ cells exhibited impaired adipogenesis, mirroring the blunted adipogenesis observed in 3T3-L1 preadipocytes with NEDD8 knockdown via siRNA and shRNA [19]. Our examination of adipogenic signaling for the first time revealed that neddylation deficiency does not affect mitotic clonal expansion. Instead, we identified deficient CREB/CEBPβ signaling as the primary factor that impairs PPARγ transcriptional activation, thereby hindering adipogenesis in NAE1^KO^ 3T3-L1 cells. Additionally, we pinpointed CUL3, rather than CUL1, CUL2, or CUL4A, as the major neddylation target regulating adipogenesis. The deficiency of CUL3 largely recapitulated the adipogenic deficiency induced by NAE1 deletion. These findings provide novel insights into the role of neddylation and its targets in mediating adipogenesis, highlighting the specific importance of CUL3 in this process. 

Previous studies have suggested that PPARγ is neddylated by the MDM2 E3 ligase, and this neddylation stabilizes PPARγ protein expression, and, thus, sustaining adipogenesis [19]. In contrast, neddylation is known to activate CUL4B–RING E3 ligase (CRL4B), which negatively regulates PPARγ by promoting its polyubiquitination and proteasomal degradation [21]. Although we did not directly assess whether PPARγ is neddylated in our study, we predominantly observed downregulation of PPARγ at the mRNA level in differentiating NAE1^KO^ cells, which was attributed to an initial defect in CREB/CEBPβ signaling. Despite the severely blunted PPARγ signaling, treatment with the PPARγ agonist pioglitazone could only minimally rescue adipogenesis. This highlights the complex role of neddylation in regulating multiple aspects of adipogenesis beyond the regulation of PPARγ signaling alone. Further research is needed to identify these additional pathways and elucidate the comprehensive mechanisms by which neddylation influences adipocyte differentiation.

Single-cell RNA-Seq data identified CUL1, CUL2, CUL3, CUL4A, and CUL5 as the primary CULs in mouse and human adipose stromal progenitor cells and adipocytes, which express minimum amounts of CUL4B, CUL7, and CUL9 [40]. In this study, we first characterized the regulation of CUL1, CUL2, CUL3, and CUL4A during adipogenesis and investigated their potential roles in regulating this process. We observed that each CUL member shows varying degrees of downregulation during adipogenesis, affecting their neddylated and/or native forms. This was in line with the overall reduction in neddylated CULs in the differentiated adipocytes. Notably, previous reports indicated an increase in free NEDD8 [19] and CUL3 neddylation levels [28] during adipogenesis, which contrasts with our findings. The discrepancy might be due to different cell systems used in the studies. 

Our data emphasizes that the intact neddylated CULs are essential for the initiation of adipogenesis despite its ultimate downregulation in mature adipocytes. Such findings, though counterintuitive, can still be plausible. In fact, inhibiting neddylation in the first two days, but not day 2 to 4, largely prevented adipogenesis, at least partially by impairing the early-stage cAMP/PKA-triggered CREB/CEBPβ/PPARγ signaling. UPS has been implicated in regulating PKA holoenzyme activity through the RING ligase Praja2-mediated degradation of the PKA regulatory subunit [41] and CHIP-mediated ubiquitination of the PKA catalytic subunit [42]. Other components of the cAMP/PKA signaling pathway have also been shown to be regulated by UPS [43]. In particular, the catalytic subunit of protein phosphatase 2A (PP2A), which dephosphorylates the PKA substrates and attenuates the signal, is targeted by CRL3 [44]. The exact mechanisms underlying the neddylation and CUL3-regulated CREB/CEBPβ/PPARγ signaling await further dissecting. 

Our data argues against CUL1 as the dominant target of neddylation in mediating adipogenesis. First, although neddylation deficiency causes dysregulated expressions of SKP1/CUL1/F-box ligases known to target cell cycle regulators [15,35,36], it did not perturb overall mitotic clonal expansion. Second, CUL1 deletion in 3T3-L1 preadipocytes did not significantly block adipogenic programs, as evidenced by similar PPARγ and PLIN1 expression levels. The minimal reduction in TG contents in the differentiated CUL1-KO adipocytes may be associated with its role in regulating lipogenesis, given that SCF E3 ligases have also been shown to regulate SREBP1c, which influences lipid droplet growth [45]. A recent study identified CUL2–APPBP2 as the ubiquitin E3 ligase determining PRDM16 protein stability by catalyzing its polyubiquitination [46]. However, we found that white adipocyte differentiation was unaffected even with at least 90% deletion of CUL2. Whether CUL2 influences brown adipogenesis through regulating PRDM16 stability remains to be further investigated. The roles of CUL4A and CUL4B in adipogenesis have been controversial. One study found that CUL4A depletion substantially promotes 3T3-L1 adipogenesis, whereas adipogenesis was markedly inhibited by CUL4B depletion [47]. Conversely, another study demonstrated that CUL4B represses adipogenesis by targeting PPARγ for ubiquitin-mediated proteasome degradation [21]. Our study identified no clear differences in 3T3-L1 adipogenesis when CUL4A was deleted by the CRISPR/Cas9 approach. The reason for the differential effects of CUL4A and CUL4B knockdown on 3T3-L1 adipogenesis in different studies remains unclear. Given the multiple distinct CRL4 complexes assembled in 3T3-L1 cells, these studies must be interpreted cautiously. The role of CUL5 in adipogenesis remains to be determined in the future.

Deletion of CUL3 almost phenocopied NAE1 deletion in impairing CREB/CEBPβ/PPARγ signaling and thus adipogenesis. This suggests that CUL3 is one of the important CULs targeted by neddylation that mediate adipocyte differentiation. The exact role of neddylation and CUL3 in mediating CREB/CEBPβ/PPARγ signaling remains mysterious. Other pathways may also contribute to the transcriptional activation of PPARγ. High RHOA/ROCK signaling through ectopic overexpression of RHOA or treatment with the RHOA agonist lysophosphatidic acid suppresses PPARγ expression and actin polymerization, and, thus, adipogenesis [39]. In our study, we observed an upregulation of RHOA in CUL3^KO^ preadipocytes. However, treating differentiating CUL3^KO^ cells with RHOA/ROCK inhibitor Y27632 failed to rescue adipogenesis in CUL3^KO^ (Figure 8), as well as NAE1^KO^ cells, arguing against RHOA as the primary target of CUL3 in mediating adipogenesis. CHOP (also called growth arrest-DNA damage-induced 153, GADD153), a dominant negative form of CEBP family members, blocks adipogenesis by delaying the activity of CEBPβ (43). CHOP has also been shown as a CRL3^KEAP1^ target [48]. However, we did not observe upregulated CHOP in CUL3^KO^ and NAE1^KO^ cells. Therefore, which CRL3 substrate contributes predominantly to adipogenesis remains to be clarified.

## 5. Conclusions

In summary, we have identified neddylation as a necessary pathway that stimulates adipogenesis. It potentially acts on CUL3 by regulating the CREB/CEBPβ/PPARγ signaling pathway to influence adipogenesis. Further research is needed to elucidate the precise mechanisms by which neddylation and CUL3 influence adipogenesis.

## Figures and Tables

**Table 1:** Guide RNA sequences targeting murine genes.

Gene Name	gRNA Sequence
**mNae1 gRNA1**	TATAGGCTGTGGGGTGATCA
**mNae1 gRNA2**	GAACCGAGCTCAAGCTGCCA
**mCul1 gRNA1**	CCAACAAACTGAGCTCCCCC
**mCul1 gRNA2**	TGCATCAGTCCAACCAAGCC
**mCul2 gRNA1**	ACTATATGGACTGCTTATAT
**mCul3 gRNA1**	GACCACTGTTATTCTTACGC
**mCul3 gRNA2**	AACTTCTCCTTTCCGCTCTC
**mCul4 gRNA1**	GCTCTACAAGCAGCTGCGCC

**Table 2:** RT-PCR primer sequences for murine (m) and human (h) genes.

Gene Name	5′ Forward Primer Sequence	3′ Reverse Primer Sequence
***mCebpa***	GAACAGCAACGAGTACCGGGTA	GCCATGGCCTTGACCAAGGAG
***mCebpb***	CAAGCTGAGCGACGAGTACA	CAGCTGCTCCACCTTCTTCT
***mPlin1***	CACCTGCGGCTGTGCTGG	CGATGTCTCGGAATTCGCT
***mPparγ2***	TCTCCTGTTGACCCAGAGCA	GTGGAGCAGAAATGCTGGAG
***mPpia***	CTGTTTGCAGACAAAGTTCCA	AGGATGAAGTTCTCATCCTCA
***mRplp0***	CGCTTTCTGGAGGGTGTCCGC	TGCCAGGACGCGCTTGTACC
***hRPLP0***	GCAATGTTGCCAGTGTCTGT	AGATGGATCAGCCAAGAAGG
***hPPARγ***	TCCATGCTGTTATGGGTGAA	ACGGAGCTGATCCCAAAGT
***hPlin1***	TCCCTCCAGACAAGGAAGAG	CATGGTCTGCACGGTGTATC

**Table 3:** Primary and secondary antibodies are used for immunoblotting.

Antibody	Supplier (Catalog No.)	RRID
**ACTB**	Sigma-Millipore (MAB1501)	RRID:AB_2223041
**AKT**	Cell signaling technology (9272)	RRID:AB_329827
**Phospho-AKT (ser473)**	Cell signaling technology (4060)	RRID:AB_2315049
**CCND1 (Cyclin D1)**	Cell signaling technology (2978)	RRID:AB_2259616
**CCND3 (Cyclin D3)**	Cell signaling technology (2936)	RRID:AB_2070801
**CDKN1B (p27)**	Cell signaling technology (3698)	RRID:AB_2077832
**CREB (48H2)**	Cell signaling technology (9197)	RRID:AB_331277
**Phospho-CREB (Ser133)**	Cell signaling technology (9198)	RRID:AB_2561044
**CUL1**	Proteintech (12895-1-AP)	RRID:AB_2086291
**CUL2**	Abcam (ab166917)	
**CUL3**	Cell signaling technology (2759)	RRID:AB_2086432
**CUL4A**	Proteintech (14851-1-AP)	RRID:AB_2261175
**ERK1/2**	Cell signaling technology (9102)	RRID:AB_330744
**Phospho-ERK1/2**	Cell signaling technology (4370)	RRID:AB_2315112
**GAPDH**	Proteintech (60004-1-IG)	RRID:AB_2107436
**NAE1**	Cell signaling technology (14321)	RRID:AB_2798448
**NEDD8**	Cell signaling technology (2754)	RRID:AB_659972
**PLIN1**	Cell signaling technology (9349)	RRID:AB_10829911
**PPARγ**	Cell signaling technology (2435)	RRID:AB_2166051
**RHOA**	Proteintech (10749-1-AP)	RRID:AB_2285104
**TUBA1A**	Proteintech (66031-1-Ig)	RRID:AB_11042766

## Data Availability

The lead contact can provide any information required to reanalyze the data reported in this article upon request.

## Supplementary Materials

The following supporting information can be downloaded at: https://www.mdpi.com/article/10.3390/cells13191654/s1, Figure S1: MLN4924 does not affect cell growth during the first 48 hours of adipocyte differentiation; Figure S2: Neddylation deficiency does not affect mitotic clonal expansion during adipocyte differentiation.
