# Supplementary material for: Neddylation and Its Target Cullin 3 Are Essential for Adipocyte Differentiation
Source: Cells. 2024 Oct 5;13(19):1654. doi: 10.3390/cells13191654 (PMC11475318; doi:10.3390/cells13191654)
Supplement: Supplementary file 1 [file cells-13-01654-s001.zip › cells-3181796-supplementary.pdf]

Supplemental Figures

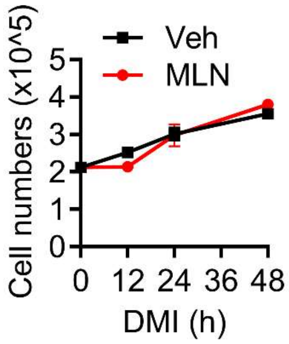

Supplemental Figure S1. MLN4924 does not affect cell growth during the first 48 hours of adipocyte differentiation. 3T3-L1 cells were grown to confluence. Two days after confluence, cells were treated with standard hormone cocktail DMI (Dexamethasone, IBMX: 3-Isobutyl-1-Methylxanthine, and Insulin) concurrently with vehicle (Veh) or 0.5 µM MLN4924 (MLN) for the first 48 hours. Cell numbers were counted at 0 (2 days after confluence), 12 h, 24 h, and 48 h post-DMI treatment (n=3). Two independent experiments.

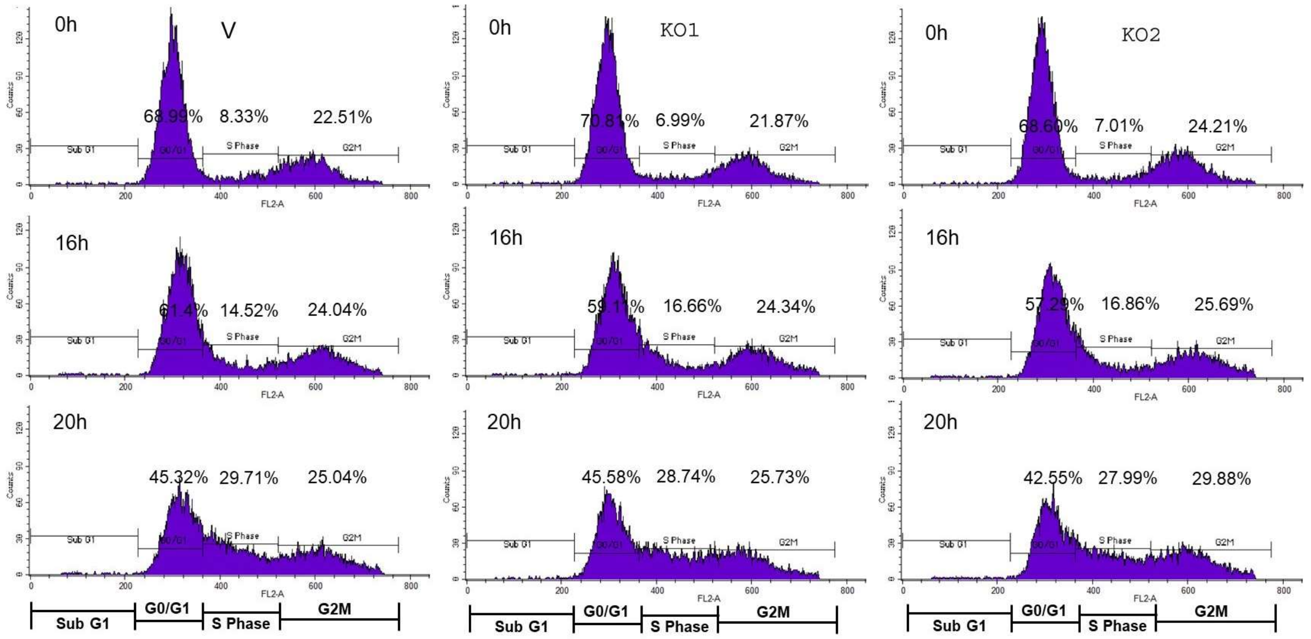

Supplemental Figure S2. Neddylation deficiency does not affect mitotic clonal expansion during adipocyte differentiation. FACS analysis of cell population at different cell cycle stages after DMI treatment for indicated times (0, 16, and 20 h) in V, NAE1 gRNA1-knockout ($NAE1^{KO1}$, KO1), NAE1 gRNA2-knockout ($NAE1^{KO2}$, KO2) 3T3-L1 cells. Two independent experiments.
